# Laevilacunaria (Mollusca, Gastropoda) in the Southern Ocean: A comprehensive occurrence dataset

**DOI:** 10.3897/BDJ.11.e111982

**Published:** 2023-10-09

**Authors:** Andreas Schmider-Martínez, Claudia S. Maturana, Yarleth Poveda, Sebastián Rosenfeld, Zambra López-Farrán, Thomas Saucède, Elie Poulin, Claudio González-Wevar

**Affiliations:** 1 Instituto de Ciencias Marinas y Limnológicas (ICML), Facultad de Ciencias, Universidad Austral de Chile, Valdivia, Chile Instituto de Ciencias Marinas y Limnológicas (ICML), Facultad de Ciencias, Universidad Austral de Chile Valdivia Chile; 2 Centro i-mar, Universidad de Los Lagos, Puerto Montt, Chile Centro i-mar, Universidad de Los Lagos Puerto Montt Chile; 3 Instituto de Biodiversidad de Ecosistemas Antárticos y Subantárticos (BASE), Santiago, Chile Instituto de Biodiversidad de Ecosistemas Antárticos y Subantárticos (BASE) Santiago Chile; 4 Cape Horn International Center (CHIC), Puerto Williams, Chile Cape Horn International Center (CHIC) Puerto Williams Chile; 5 Universidad Austral de Chile, Valdivia, Chile Universidad Austral de Chile Valdivia Chile; 6 Centro de Investigación Gaia‑Antártica, Universidad de Magallanes, Punta Arenas, Chile Centro de Investigación Gaia‑Antártica, Universidad de Magallanes Punta Arenas Chile; 7 Centro Fondap de Investigación en Dinámica de Ecosistemas Marinos de Altas Latitudes (IDEAL), Universidad Austral de Chile, Valdivia, Chile Centro Fondap de Investigación en Dinámica de Ecosistemas Marinos de Altas Latitudes (IDEAL), Universidad Austral de Chile Valdivia Chile; 8 Biogéoscience, UMR CNRS 6282, Université de Bourgougne 6, Dijon, France Biogéoscience, UMR CNRS 6282, Université de Bourgougne 6 Dijon France

**Keywords:** Littorinids, *Laevilacunaria*, Laevilitorininae, Antarctic, sub-Antarctic, Southern Ocean

## Abstract

**Background:**

The present dataset is a compilation of georeferenced occurrences of the littorinid genus *Laevilacunaria* Powell, 1951 (Mollusca, Gastropoda) in the Southern Ocean. Occurrence data were obtained from field expeditions (Antarctic and sub-Antarctic sampling) between 2015 and 2022, together with a review of published literature including records from 1887 to 2022. Three *Laevilacunaria* species have been recorded from the Southern Ocean: *Laevilacunariabennetti*, *L.antarctica* and *L.pumilio*.

**New information:**

The present dataset includes 75 occurrences, representing the most exhaustive database of this Antarctic and sub-Antarctic littorinid genus. The publication of this data paper was funded by the Belgian Science Policy Office (BELSPO, contract n°FR/36/AN1/AntaBIS) in the Framework of EU-Lifewatch as a contribution to the SCAR Antarctic biodiversity portal (biodiversity.aq).

## Introduction

Species-distribution knowledge is a key parameter to understand the composition and behaviour of communities in different environments. Many studies, based on species distributions, allow us to understand the evolutionary pathways and biogeographical patterns, as well as the processes and mechanisms associated with their origins and diversification (Pearse et al. 2009, Allcock and Strugnell 2012, Fraser et al. 2012, González-Wevar et al. 2013, Fraser et al. 2014, González-Wevar et al. 2016, González-Wevar et al. 2021, Rosenfeld et al. 2022). A number of initiatives are currently facing the challenge to record species and their distributions, including the Census of Marine Life (http://www.coml.org/about-census/), whose results were compiled in the Biogeographic Atlas of the Southern Ocean (De Broyer et al. 2014). This is undoubtedly a difficult scientific task, but several initiatives are trying to face it through freely available platforms, such as the Ocean Biodiversity Information System (OBIS) and Global Biodiversity Information Facility (GBIF), where different kinds of collectors including museums, herbaria and researchers can offer their respective datasets. The laboratory of *Genómica y Ecología Molecular Antártica y sub-Antártica* (LAGEMAS), located at the Universidad Austral de Chile, is also making an effort to nurture and update this open and free-access dataset of occurrence information on higher latitude littorinids for future studies in distribution, biogeography and modelling.

A baseline survey of Antarctic biodiversity indicated that molluscs are one of the most abundant and widely distributed groups and have been recorded in 85% of intertidal localities, more than arthropods (55%) or macroalgae (44%) (Griffiths & Waller 2016). The most ubiquitous mollusc species were the patellogastropod *Nacellaconcinna* (Antarctic limpet) and the littorinid *Laevilitorinacaliginosa*, present in 56 and 45 of the 98 areas studied by Griffiths and Waller (2016), respectively.

The family of the intertidal gastropods Littorinidae Children, 1834, commonly known as periwinkles, includes more than 200 species in three subfamilies; Lacuninae, Laevilitorininae and Littorininae (Bouchet et al. 2005). These small gastropod snails are very abundant in shallow intertidal rocky ecosystems worldwide and particularly in temperate and tropical areas. Littorinids are some of the most extensively used model organisms in ecology (Eschweiler et al. 2009), evolution (Johannesson 2003, Reid et al. 2012), speciation (Williams et al. 2003), physiology (McMahon 2001) and reproduction (Johannesson et al. 1995). They have also been used for micro-evolutionary studies including natural selection and genetic differentiation (Reid et al. 2006) and macro-evolutionary studies, such as adaptive radiation and historical biogeography (Reid 1996, Williams et al. 2003, González‐Wevar et al. 2022). Most of the studies in littorinids have been done in temperate groups and little is known about the origin and evolutionary relationships of higher latitude Antarctic and sub-Antarctic genera.

The Southern Ocean includes three littorinid genera: *Pellilitorina* Pfeffer, 1886, *Laevilacunaria* Powell, 1951 and *Laevilitorina* Pfeffer, 1886. The genus *Pellilitorina* belongs to the subfamily Lacuninae Gray, 1857, while *Laevilitorina* and *Laevilacunaria* are the extant members of the subfamily Laevilitorininae.

The genus *Laevilacunaria* includes three species: *L.bennetti* (Preston, 1916), *L.antarctica* (Martens, 1885) and *L.pumilio* (E. A. Smith, 1877). *Laevilacunariaantarctica* was originally described under the name *Lacunaantarctica* from South Georgia (Martens 1885). Similarly, *L.bennetti* was described as *Pellilitorinabennetti* from the South Shetland Islands, Antarctic Peninsula (Preston 1916) and *L.pumilio* was described as *Hydrobiapumilio* from the Kerguelen Islands (E. A. Smith 1877). Subsequently, based on morphological characteristics of the shell and radula, Powell (1951) described the genus *Laevilacunaria* and placed these three species in the genus. The distribution of *L.antarctica* includes hard rocky-bottom ice-free sublittoral ecosystems across maritime Antarctic, including the Antarctic Peninsula, South Shetland Islands, South Orkney Islands, Signy Island and South Georgia (Picken 1979, Iken 1999, Engl 2012, Amsler et al. 2015, Aghmich et al. 2016, Martin et al. 2016) The distribution of *L.bennetti* also includes rocky ice-free sublittoral ecosystems across the Antarctic Peninsula, South Shetland Islands and the Palmer Archipelago (Picken 1980, Amsler et al. 2015). Finally, *L.pumilio* was described in the sub-Antarctic Kerguelen Archipelago, but it has also been reported at the Bellinghausen Sea in the Antarctic Peninsula (Rosewater 1970, Ivanova and Grebelnyi 2017).

The Antarctic *Laevilacunaria* species (*L.antarctica* and *L.bennetti*) exhibit narrow bathymetric distributions, compared to other Antarctic marine invertebrates, that range from the upper intertidal to 90 m depth, with maximum abundance recorded at 12 m depth (Reid et al. 2012). Both species are highly abundant and have ecological importance in almost all marine ecosystems across the Antarctic Peninsula. As recorded in most Antarctic marine invertebrates, *Laevilacunaria* species exhibit marked seasonal, spatial and growth variability throughout their distributions. According to Picken (1979) and Amsler et al. (2015), the highest abundances of *L.antarctica* are found associated with macroalgae like *Desmarestia anceps*, *Desmarestia menziesii*, *Pseudophycodrys* sp. and over the fronds of *Plocamium*, *Leptosarca*, *Sarcopeltis*, *Ascoseira*, Himantothallus, *Pantoneura* sp. and *Phyllophora* sp., where they graze and reproduce. However, ecological studies suggest that *L.antarctica* does not feed on *Ascoseira mirabilis*, *Phaeurus antarticus* or *Himantothallusgrandifolius* (Iken 1999). According to Iken (1999), *L.antarctica* feeds two thirds on epiphytic diatoms and a third on macroalgae. This is reflected in the taenioglosa-type radula of the species, a very plastic tool that allows it to feed on a wide spectrum of algae (Reid 1989, Iken 1999). Information concerning the species *L.bennetti* and *L.pumilio* is scarce; most of the records and references are associated with *L.antarctica*.

The present review documents the state of knowledge of the genus *Laevilacunaria* and provides an updated database for the Southern Ocean.

## General description

### Purpose

This dataset was created within the framework of the undergraduate thesis of the first author and part of the main research of the principal investigator. The main objective of the dataset is to build a baseline of the geographic distribution as a complement to the research on the biogeography of littorinids that is currently being carried out in the Laboratorio de Genomica y Ecología Molecular Antártica y Subantártica.

## Project description

### Title

Antarctic and sub-Antarctic littorinid database

### Personnel

Andreas Schmider-Martínez, Claudia Maturana, Sebastián Rosenfeld, Claudio González-Wevar

### Study area description

This study is centred on Antarctic and sub-Antarctic areas between latitudes 45°S and 69ºS, including different provinces of the Southern Ocean. It extends around 24,000 km from east to west and 8,000 km from north to south and covers an area of around 192 million km^2^. The objective of this study is to integrate the most complete database of species occurrences for the Antarctic and sub-Antarctic genus *Laevilacunaria* across this area.

### Design description

The present study provides a specific level dataset of *Laevilacunaria* including 75 records, using a combination of data collected during recent Antarctic campaigns and literature. This review is the most updated and exhaustive database on this important Antarctic littorinid genus and will be a base for future biogeographic and/or phylogeographic analyses.

### Funding

Regular FONDECYT 1210787 Project, Initiation FONDECYT 11140087 project, Millennium Institute Biodiversity of Antarctic and Subantarctic Ecosystems (Mi-BASE) - Instituto Milenio ICN2021_002, Project Genomic Antarctic Biodiversity (GAB) - PIA CONICYT ACT172065, Research Center Dynamics of High Latitude Marine Ecosystems - Fondap-IDEAL 15150003, Postdoc FONDECYT 3210063, INACH DG_10-22 and Regular INACH RG_18-17 project.

## Sampling methods

### Study extent

The compilation of records for the database of the genus *Laevilacunaria* in the Southern Ocean was obtained by two different types of sources: 1) field sampling data obtained during the Chilean Antarctic Scientific Expedition (ECA) between the years 2015 (ECA51) and 2022 (ECA59), together with records from Crozet and Kerguelen Islands through the PROTEKER project (an Underwater Observatory at Kerguelen) supported by the French Polar Institute; and 2) literature review, museums and cruise reports. The search for public literature included scientific manuscripts and books concerning *Laevilacunaria* and species that were once included in the genera *Laevilitorina, Pellilitorina, Hydrobia* and *Lacuna*, which now belong to *Laevilacunaria*.

### Sampling description

**Figure 1.**
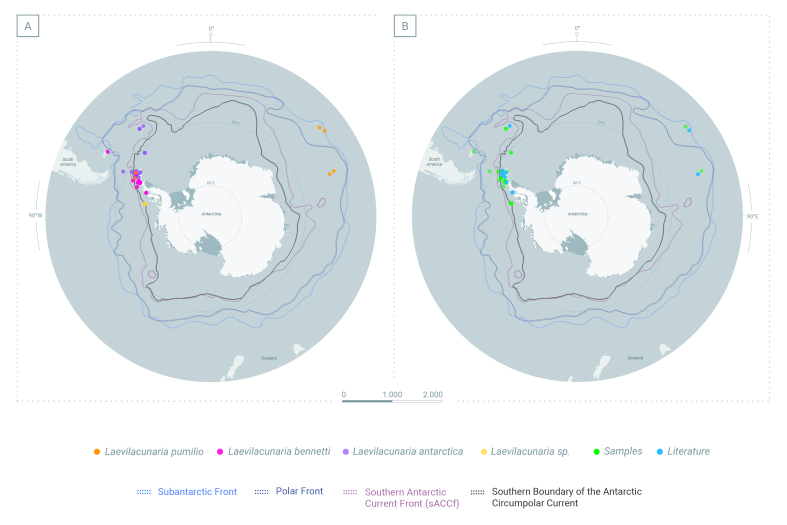
Distribution of all records of the genus *Laevilacunaria* in the Southern Ocean (SO). **A** Distribution of the three different species of *Laevilacunaria* in the SO; **B** Distribution of the specimen of the genus *Laevilacunaria* reported in the Southern Ocean showing the origin of the sources; literature and sampling.

**Figure 2.**
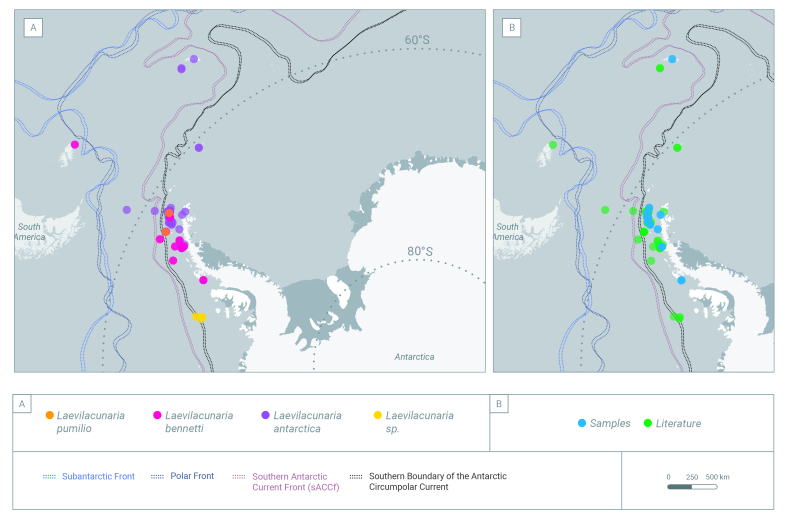
The distribution of *Laevilacunaria* across the Antarctic Peninsula, showing: **A** Distribution of the three different species of *Laevilacunaria*; **B** Distribution of the individuals of the genus *Laevilacunaria* reported in the Southern Ocean showing the origin of the sources; literature and sampling.

**Figure 3.**
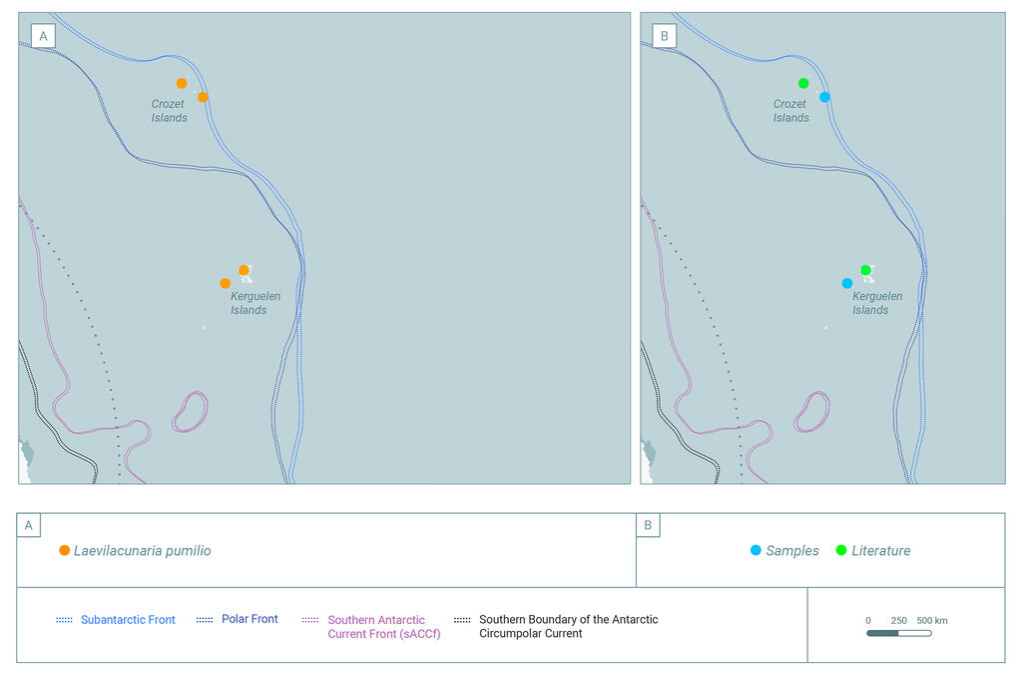
The distribution of *Laevilacunaria* in the Crozet Islands and Kerguelen Islands, showing: **A** Distribution of the three different species of *Laevilacunaria*; **B** Distribution of the individuals of the genus *Laevilacunaria* reported in the Southern Ocean showing the origin of the sources; literature and sampling.

Occurrence data were compiled from Antarctic campaigns at different localities across the Southern Ocean (Fig. 1 and Table 1) including the South Shetland Islands (Penguin, Greenwich, King George, Livingstone, Robert, Deception), the west Antarctic Peninsula (Marguerite Bay, Isabel Riquelme Island, Doumer Island) (Fig. 2), the sub-Antarctic South Georgia Island during 2015-2022 and Crozet and Kerguelen Archipelagos (Fig. 3). The sampling was the result of an international and national collaboration network.

An extensive revision of the genus *Laevilacunaria* was done in the ISI web of Knowledge searching for the current scientific name of the group: *Laevilacunaria* and species of *Laevilitorina*, *Pellilacunella*, *Pellilitorina*, *Lacuna* and *Hydrobia*, which now are included in Laevilacunaria, as well as other names like *Laevilacunaria/Pellilitorinabrandsfieldensis* and *Laevilacunariabennetti* which were also included in the examination. In order to obtain the distribution of the species, we collected information from manuscripts and books where *Laevilacunaria* was mentioned and included localities, dates, coordinates, expeditions, depths and years (Rosewater 1970, Arnaud and Bandel 1976, Picken 1979, Picken 1980, Iken 1999, Amsler et al. 2015, Simone 2018). All papers that mention the presence of *Laevilacunaria* using a quote were noted, but not necessarily included in the database, since they did not include geographical coordinates, locality description or were duplications of other references.

### Quality control

Duplicate data were combined into a single record to construct a unified database. To evaluate the quality of the filtered dataset, all records were checked for mismatches between reported geographic location and the associated metadata. All taxonomic records were included for the geographic distribution.

### Step description

Occurrences are presence-only data for two different sampling methodologies: 1) individual collection of *Laevilacunaria* by hand in intertidal pools during low tide periods and 2) SCUBA diving from 0 - 15 m depth where we collected: a) individuals directly by hand and/or b) substrate (sediment, macroalgae) and the associated fauna (Rosenfeld et al. 2022). All individuals were immediately preserved in ethanol (95%) for further molecular and morphological analyses at the LAGEMAS laboratory, Universidad Austral de Chile, Valdivia. Geographical coordinates were recorded using GPS for each sampling site.

## Geographic coverage

### Description

The genus *Laevilacunaria* is represented across the whole Southern Ocean and the adjacent Islands (Fig. 1): South Shetland Islands, Signy Island, Anvers Island, Elephant Island, archipelagos like the Archipelagos of Palmer, Archipelagos of Melchior and along the Antarctic Peninsula (Fig. 2), the sub-Antarctic Islands of Crozet, Kerguelen (Fig. 3) and South Georgia.

**Figure 4.**
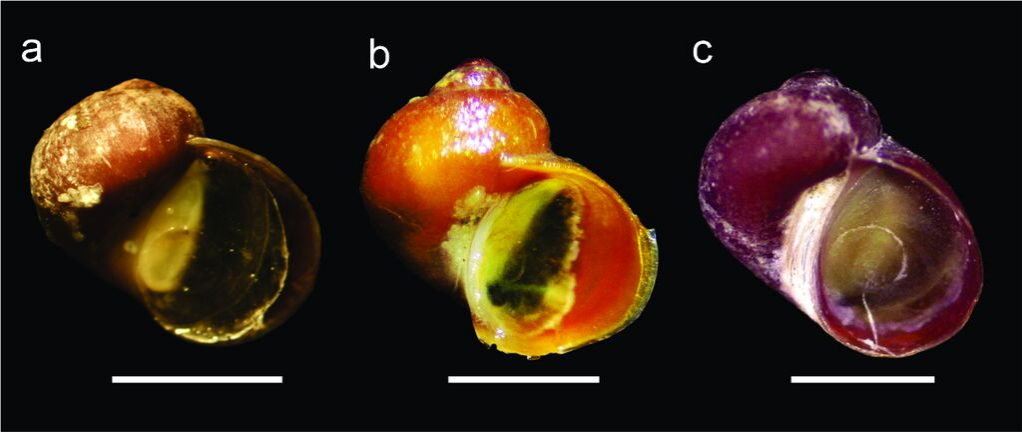
*Laevilacunaria* spp. **a** *Laevilacunariaantarctica* (scale bar 2.5 mm); **b** *Laevilacunariabennetti* (scale bar 2 mm); **c** Laevilacunariapumilio (scale bar 1 mm). Photograph (a) from Rosenfeld et al. (2022), (b) and (c) by Sebastián Rosenfeld.

*Laevilacunariaantarctica*:

Individuals of *L.antarctica* were found on intertidal rocky ecosystems across the Antarctic Peninsula and the South Shetland Islands (Fig. 4a).

*Laevilacunariabennetti*:

*Laevilacunariabennetti* (Fig. 4b) was less abundant than *L.antarctica* and was found only at Fildes Bay (King George Island), Discovery Bay (Greenwich Island), South Bay (Doumer Island) and Avian Island (Marguerite Bay). The described distribution for this species includes Anvers Island, along the Archipelago of Palmer and the South Shetland Islands, the Bransfield Strait and the Schollaert Channel.

*Laevilacunariapumilio*:

The elusive species *L.pumilio* (Fig. 4c) has been recorded in literature at the type locality, the Kerguelen Archipelago, Crozet Islands, with dubious records at Fildes Bay (King George Island, South Shetland Islands) and the Bellinghausen Sea. During the recent Proteker expedition, our French collaboration team recorded five *L.pumilio* from Crozet Islands and one from the Kerguelen Islands.

### Coordinates

-70 and -46 Latitude; -78 and 70 Longitude.

## Taxonomic coverage

### Description

The complete record of the database in *Laevilacunaria* contains 75 occurrences, including the three species of the genus across their distributions in the Southern Ocean. The literature review includes 64 records of species of *Laevilacunaria*, of which 57 were used for the geographic distribution studies and seven were not included because they were quotes of other mentions. Of the 57 records, 41 were *L.antarctica*; 10 *L.bennetti*; three *L.pumilio* and three *Laevilacunaria* as a genus. The sampling records contain a total of 261 individuals, of which 209 were *L.antarctica*, 46 *L.bennetti* and six *L.pumilio*. The time coverage of the dataset starts in 1877 (E. A. Smith 1877) and ends in 2022 with samples collected during ECA 58.

The present dataset is the most exhaustive and updated list of available occurrences and material of *Laevilacunaria* (Littorinidae) in the Southern Ocean. This collection provides information about the occurrence of three *Laevilacunaria* species: *Laevilacunariaantarctica* (Fig. 4a), *L.bennetti* (Fig. 4b) and *L.pumilio* (Fig. 4c). Occurrence distribution is shown in Fig. 1.

### Taxa included

**Table:** 

Rank	Scientific Name	
genus	*Laevilacunaria*	
species	*Laevilacunariaantarctica*	
species	*Laevilacunariabennetti*	
species	*Laevilacunariapumilio*	

## Traits coverage

### Taxonomic identification

All newly-collected *Laevilacunaria* specimens identified in this study (Fig. 4) showed morphological characteristics corresponding to those described in literature (Reid 1989, Engl 2012). Most of the studies in *Laevilacunaria* have been focused on *L.antarctica*, while *L.bennetti* and *L.pumilio* have not been considered. In fact, neither of these species has a particular study and *L.pumilio* has only been cited in its original description.

## Temporal coverage

### Notes

1887-03-01 through 2022-02-08

## Collection data

### Collection name

Coleccion de Invertebrados del Laboratorio de Genómica y Ecologia Molecular de la Universidad Austral de Chile

### Collection identifier

urn:UACh:LAGEMAS:Inv:Mol

### Parent collection identifier

LAGEMAS

### Specimen preservation method

alcohol 95%

## Usage licence

### Usage licence

Creative Commons Public Domain Waiver (CC-Zero)

### IP rights notes

This work is licensed under a Creative Commons Attribution (CC-BY 4.0) License.

## Data resources

### Data package title

Laevilacunaria (Mollusca, Gastropoda) in the Southern Ocean: A comprehensive occurrence dataset.

### Resource link

https://doi.org/10.15468/eequqq

### Alternative identifiers

https://www.gbif.org/dataset/c70094e2-7607-42da-8fb2-76669ac5c1ac; http://gbif-chile.mma.gob.cl/ipt/resource?r=genus_laevi_base

### Number of data sets

1

### Data set 1.

#### Data set name

Laevilacunaria (Mollusca, Gastropoda) in the Southern Ocean: A comprehensive cccurrence dataset.

#### Data format

Darwin Core

#### Description

All data collected for analysis reported here, including the study samples and literature, have been deposited and incorporated in the available information on the Global Biodiversity Information Facility (Schmider-Martínez et al. 2023).

**Data set 1.** 

Column label	Column description
occurrenceID	Unique identifier for each occurrence per taxa.
occurrenceStatus	The statement about the presence of the Taxon at the given Location.
materialSampleID	The unique identifier for the MaterialSample.
collectionID	The identifier for the collection or dataset from which the record was derived.
taxonRank	The taxonomic rank of the most specific name in the scientificName.
kingdom	The full scientific name of the kingdom in which the taxon is classified.
phylum	The full scientific name of the phylum in which the taxon is classified.
class	The full scientific name of the class in which the taxon is classified.
order	The full scientific name of the order in which the taxon is classified.
family	The full scientific name of the family in which the taxon is classified.
genus	The full scientific name of the genus in which the taxon is classified.
scientificName	The full scientific name in the lowest level taxonomic rank that was determined.
specificEpithet	The name of species epithet of the scientificName.
scientificNameAuthorship	The authorship information for the scientificName formatted according to the conventions of the applicable nomenclaturalCode.
scientificNameID	The identifier for the nomenclatural details of a scientific name.
country	The name of the country or major administrative unit in which the Location occurs.
countryCode	The standard code for the country in which the Location occurs following the best practice using an ISO 3166-1-alpha-2 country code.
locality	The specific description of the place in which the collection was made.
island	The name of the island on which the Location occurs.
decimalLongitude	The geographic longitude in decimal degrees of the geographic centre of a Location. Positive values are east of the Greenwich Meridian, negative values are west of it.
decimalLatitude	The geographic latitude in decimal degrees of the geographic centre of a Location. Positive values are north of the Equator, negative values are south of it.
verbatimCoordinates	The verbatim original spatial coordinates of the Location.
coordinateUncertaintyInMetres	The horizontal distance in metres from the given decimalLatitude and decimalLongitude describing the smallest circle containing the whole of the Location. We used the reasonable lower limit on or after 01-05-2020- of a GPS.
eventDate	The date-time when the event was recorded. We used best practice using the ISO 8601:1:2019.
year	The four-digit year in which the Occurrence was recorded, according to the Common Era Calendar.
month	The integer month in which the Occurrence was recorded.
minimumDepthInMetres	The lesser depth below the local surface in metres.
maximumDepthInMetres	The greater depth below the local surface in metres.
individualCount	The number of individuals present at the time of the Occurrence if it was countable.
basisOfRecord	The specific nature of the data record. We used the recommended best practice of one of the Darwin Core classes.
type	The nature of the resource. We used the recommended best practice of one of the Darwin Core classes.
preparations	A list concatenated and separated of preparations and preservation methods for the specimen.
recordedBy	A person or a list of names of people responsible for recording the original Occurrence.
identifiedBy	A person or a list of names of people responsible for recording the original Occurrence.
samplingProtocol	Descriptions of the methods used during the sampling Event.
associatedReferences	A list concatenated and separated of bibliographic reference of literature associated with the Occurrence.
habitat	A category or description of the habitat in which the Event occurred.
associatedMedia	A list concatenated and separated of publications associated with the Occurrence.
rightsHolder	The laboratory owning and managing rights over the resource.
organismRemarks	Comments about the nomenclatural history of the specie.

## Additional information

### Discussion

The compilation of species distributions has increased in the last few years with different projects like the SCAR projects, National Antarctic Science projects, the Census of Marine Life (http://www.coml.org/about-census/), the Millennium Institute of Antarctic and sub-Antarctic Biodiversity (Mi-BASE) and the Biogeographic Atlas of the Southern Ocean (De Broyer et al. 2014), amongst others. Understanding species distribution patterns is pivotal for other scientific areas including evolution, biogeography, ecology and modelling studies; to have better knowledge of these topics would permit us to interpret the origins and the evolutionary routes and, thus, allow us to predict better future scenarios under current climate change. This study represents a contribution to improve our knowledge of biodiversity by increasing the open access database for future biogeographic and species modelling for Southern Ocean littorinids and particularly for *Laevilacunaria*.

### Literature remarks

This study unifies the knowledge of biodiversity and geographical distribution records for *Laevilacunaria* using two different types of species reports (literature and sampling). The other two recognised species of the genus have only been mentioned in a couple of studies (including their original descriptions), which are not directly studies of marine invertebrates. Since then, *L.pumilio* only has been mentioned in three other studies: 1) Rosewater (1970), a summary of the family of the Littorinidae in the Indian Ocean; 2) Cantera and Arnaud (1984), a gastropod study and mention of *L.pumilio* by Crozet; 3) Ivanova and Grebelnyi (2017), studying the stomach content of anemones where *L.pumilio* was identified with a photograph. However, as previously stated, the actual presence of the Kerguelenian species *L.pumilio* in Antarctica requires further confirmation. *Laevilacunariapumilio* is the only species of *Laevilacunaria* for which no description of radula morphology has been made. *Laevilacunariabennetti* has been mentioned on five occasions since the first description of Preston (1916): 1) Powell (1951) showed several locations of the distribution of *L.bennetti* across the Antarctic; 2) Rosewater (1970), a summary of the family of the Littorinidae in the Indian Ocean; 3) Picken (1980), explaining the reproductive patterns of *L.bennetti*, the first study delivering information concerning the species; 4) Taylor and Reid (1990) and 5) Amsler et al. (2015), mentioned the presence of the species. By contrast, *L.antarctica* has been mentioned in more than 36 studies since the description of E. van Martens (1885) and has been included in at least 10 direct studies (Pfeffer 1886). The species is mentioned in almost all intertidal studies of the Antarctic Peninsula and the adjacent islands. There have been several studies about feeding strategy (Picken 1979, Reid 1989, Iken 1999, Amsler et al. 2015), the reproductive cycle (Picken 1979, Picken 1980), morphology (Engl 2012), radula morphology (Powell 1951, Arnaud and Bandel 1976, Simone 2018) and population genetics (González‐Wevar et al. 2022), amongst others. *L.antarctica* is commonly cited in published literature and studies due to its abundance in the majority of Antarctic intertidal areas, while *L.bennetti* is mentioned less frequently. A plausible explanation for this bias is the morphological similarity of the two species (Powell 1951) and also the fact that *L.bennetti* mainly inhabits subtidal environments, while *L.antarctica* is commonly found from intertidal. Several studies mention that *L.antarctica* has been found in samples from 0-30 m depth, while *L.bennetti* is described as a more subtidal species with distribution between 6 and 16 m; field observation confirm this suggestion.

Another important mention is the transition of the species of the genus *Laevilacunaria. Laevilacunariaantarctica* underwent a taxonomic revision, transitioning from various genera. One reason for this is the synonymisation of the species *L.brandsfieldensis* with *L.antarctica*. *Laevilacunariaantarctica* was originally classified under the genus *Lacuna*, but was reclassified under *Laevilacunaria*, while *L.brandsfieldensis*, initially described as *Pellilitorina*, was later synonymised under *Laevilacunaria*. *Laevilacunariapumilio* underwent a reclassification, moving from the genus *Hydrobia* to *Laevilitorina* and subsequently to *Laevilacunaria*. *Laevilacunariabennetti* was transferred from *Pellilitorina* to *Laevilitorina* and ultimately to *Laevilacunaria*.

### Distribution patterns

This study compiled the knowledge of the different types of species records (published literature and sampling) to establish the distribution patterns of the genus *Laevilacunaria*. The limits seem to be the adjacent sub-Antarctic islands; South Georgia Islands, Signy Island, Trinity Island, Kerguelen Island and Crozet Island. The majority of occurrence records appear to be the West Antarctic Peninsula part of the Antarctic Peninsula with its nearby Islands (Elephant Island, South Shetland Islands, Deception Island, Anvers Island amongst others) and archipelagos (Palmer Archipelago); only a few studies indicate its presence in East Antarctica. The reason could be the contrast in the ease of access to the intertidal between the western and eastern parts of the Antarctic continent.

To summarise, the compilation cannot encompass the whole distribution of the genus *Laevilacunaria*, but brings an important update to previously-published occurrences in a thorough single database. Future studies in the three recognised species of the *Laevilacunaria* are required to resolve the systematic and biographics at macro- and micro-evolutionary scales. This study gives a basis for diverse molecular studies to reconstruct the evolutionary roads of dispersion and speciation of the genus across the Southern Ocean.

## Figures and Tables

**Table 1.** Field campaigns during 2015–2022 identifying which littorinids of the dataset were collected at each locality.

**Locality**	**Island**	**Species**	**Nº specimens**	**Date / ECA**	**ver.Coordinates**
Fildes Bay	King George Island	*L.antarctica* *L.bennetti*	13 20	2022/ECA59 2022/ECA59	62°12' S | 58°57' W
Admiralty Bay	King George Island	*L.antarctica*	10	2019/ECA56	62°12' S | 58°57' W
Pingüino Island	King George Island	*L.antarctica*	16	2020/ECA57	62°06' S | 77°52' W
Hannah Point	Livingston Island	*L.antarctica*	10	2017/54	62°39' S | 60°38' W
Coppermine Peninsula	Robert Island	*L.antarctica*	29	2017/ECA54	62°22' S | 59°42' W
Deception Island	Deception Island	*L.antarctica*	26	2017/ECA54	62°57' S | 60°40' W
Discovery Base	Greenwich Island	*L.antarctica* *L.bennetti*	48 13	2021/ECA58 2020/ECA58	62°28' S | 59°37' W
South Bay	Doumer Island	*L.antarctica* *L.bennetti*	22 8	2017/ECA54 2021/ECA58	64°52' S | 63°35' W
Isabel Riquelme Island	Ant. Peninsula	*L.antarctica*	7	2019/ECA56	63°19' S | 57°53' W
Trinity Island	Trinity Island	*L.antarctica*	5	2020/ECA58	63°47' S | 60°44' W
South Georgia	South Georgia	*L.antarctica*	2	2021/ECA59	54°26' S | 36°33' W
Avian Island	Marguerite Bay	*L.antarctica* *L.bennetti*	21 5	2017/ECA54 2019/ECA56	67°46' S | 68°52' W
Kerguelen Islands	Kerguelen Archilepagos	*L.pumilio*	1	2021/PROTEKER	46°24’ S| 51°52’ E
Crozet Islands	Crozet Island	*L.pumilio*	5	2021/PROTEKER	49°21’ S| 70°13’ E
